# Loss of Ensemble Segregation in Dentate Gyrus, but not in Somatosensory Cortex, during Contextual Fear Memory Generalization

**DOI:** 10.3389/fnbeh.2016.00218

**Published:** 2016-11-07

**Authors:** Marie Yokoyama, Naoki Matsuo

**Affiliations:** ^1^Career-Path Promotion Unit for Young Life Scientists, Kyoto UniversityKyoto, Japan; ^2^Department of Molecular and Behavioral Neuroscience, Graduate School of Medicine, Osaka UniversityOsaka, Japan; ^3^The Hakubi Center for Advanced Research, Kyoto UniversityKyoto, Japan

**Keywords:** hippocampus, memory engram, mice, context, PTSD

## Abstract

The details of contextual or episodic memories are lost and generalized with the passage of time. Proper generalization may underlie the formation and assimilation of semantic memories and enable animals to adapt to ever-changing environments, whereas overgeneralization of fear memory evokes maladaptive fear responses to harmless stimuli, which is a symptom of anxiety disorders such as post-traumatic stress disorder (PTSD). To understand the neural basis of fear memory generalization, we investigated the patterns of neuronal ensemble reactivation during memory retrieval when contextual fear memory expression is generalized using transgenic mice that allowed us to visualize specific neuronal ensembles activated during memory encoding and retrieval. We found preferential reactivations of neuronal ensembles in the primary somatosensory cortex (SS), when mice were returned to the conditioned context to retrieve their memory 1 day after conditioning. In the hippocampal dentate gyrus (DG), exclusively separated ensemble reactivation was observed when mice were exposed to a novel context. These results suggest that the DG as well as the SS were likely to distinguish the two different contexts at the ensemble activity level when memory is not generalized at the behavioral level. However, 9 days after conditioning when animals exhibited generalized fear, the unique reactivation pattern in the DG, but not in the SS, was lost. Our results suggest that the alternations in the ensemble representation within the DG, or in upstream structures that link the sensory cortex to the hippocampus, may underlie generalized contextual fear memory expression.

## Introduction

Memories are not immutable. The details of contextual or episodic memories are often lost and generalized (Houston et al., 1999; Biedenkapp and Rudy, 2007; Wiltgen and Silva, 2007; Lacy and Stark, 2013), which may underlie the formation of semantic memories or schemas (Lambon Ralph and Patterson, 2008). Moreover, adequate generalization of fear memory may contribute to the generation of adaptive responses to similar situations that predict danger (Kheirbek et al., 2012; Dunsmoor and Paz, 2015), since it is very rare to encounter exactly the same fear-inducing circumstances repeatedly in the real world. Thus, an ability to generalize memory is crucial for animals to adapt to ever-changing environments. On the other hand, excessive generalization of fear memory can cause maladaptive fear responses to harmless stimuli or situations bearing similar aspects of a learned threat, which is a symptom of anxiety disorders such as post-traumatic stress disorder (PTSD; Kheirbek et al., 2012; Mahan and Ressler, 2012; Lissek et al., 2014). Elucidating the underlying mechanism of memory generalization would thus yield important insights regarding the pathophysiology of PTSD, as well as a better understanding of the neural basis of dynamic memory processing in the brain. Recent human neuroimaging research such as fMRI has suggested several brain structures whose activity is enhanced or decreased related with generalized fear memory expression (Dunsmoor and Paz, 2015). These studies may identify brain areas involved in the retrieval of generalized fear memory, but do not address the neural basis supporting fear memory generalization.

Contextual fear conditioning is widely used for studying the mechanisms underlying learning and memory, and for the understanding and treatment of fear-related disorders such as PTSD (Fanselow and Poulos, 2005; Johansen et al., 2011; Parsons and Ressler, 2013). When animals experience an aversive event (unconditioned stimulus: US), they acquire a fear memory associated with the specific context of the event (Maren et al., 2013). Re-exposure to the same context alone evokes conditioned fear responses, such as freezing. In generalization, a perceptually similar but distinct context also elicits fear responses, implying that the balance between contextual discrimination and generalization is a crucial aspect of behavioral output, in this case the expression of fear. Thus, characterizing the contextual memory representations or processing in the brain during fear memory retrieval could provide powerful insights into the neural regulation of generalized fear expression.

A specific subset of cells that was activated during a given time window can be tagged via a transgenic mouse system that incorporated a combination of an activity-dependent *c-fos* gene promoter and a tetracycline-inducible expression system (Reijmers et al., 2007; Matsuo et al., 2008). Activities of the tagged ensembles of neurons during contextual fear learning using this system have been shown to be sufficient and necessary for the contextual fear memory expression (Garner et al., 2012; Liu et al., 2012; Cowansage et al., 2014; Tanaka et al., 2014; Matsuo, 2015; Ohkawa et al., 2015; Yoshii et al., 2017). These studies also suggested that a part of the neuronal ensemble that was activated during the initial memory encoding becomes reactivated when the memory is retrieved. This study was designed to utilize this transgenic system, by visualizing specific neuronal ensembles activated by contextual fear memory encoding and during memory retrieval, with the aim of determining the level to which activities overlap after contextual fear memory is generalized at the cellular ensemble level.

## Materials and Methods

### Mice

The generation of cfos-transactivator (tTA) × tetO-tau lacZ double transgenic mice has been described previously (Reijmers et al., 2007). The double transgenic mice were backcrossed to C57BL/6J mice and maintained as heterozygotes. All mice were bred in social groups (2–5 mice per cage), provided with food and water *ad libitum*, and fed with a doxycycline (Dox) diet (50 mg/kg chow) from pregnancy. After behavioral treatment (fear conditioning or home cage (HC)) during the off-Dox time window, they were switched to a higher concentration of Dox diet (1 g/kg chow) to quickly suppress further expression of tau-lacZ induced by unrelated behavioral stimuli. Mice that were 11–24 weeks old at the onset of experiments were used. All procedures were approved and conducted in accordance with guidelines of Kyoto University and Osaka University on the care and use of laboratory animals.

### Fear Conditioning

All behavioral experiments were conducted during the light period of the light/dark cycle. At the start of the experiments, the mice were individually housed and subjected to handling sessions for 3 days. For contextual fear conditioning, the mice were placed in a novel rectangular chamber (context A: 25 cm × 33 cm × 28 cm) with white plastic side walls, a transparent plastic top, and front and rear walls with a stainless steel grid floor under a 100 lux light in a sound-proof room (O’ Hara and Co., Ltd). Three foot-shocks (2 s, 0.75 mA) were administered at time points of 208, 298 and 388 s after the animals were placed in the chamber. They were returned to their HC 30 s after the final shock. For the memory retrieval test, the mice were placed in the conditioned context (context A) or in a novel context (context B) for 180 s. For context B, the mice were placed in a chamber consisting of a checkered cylindrical wall and a transparent plastic top with a flat plastic floor under a 50 lux light. For context C, mice were placed in a novel triangular chamber made of opaque white Plexiglas (33 cm × 29 cm × 40 cm) with a flat plastic floor with a lemon oil scent under a 20 lux red light in a different sound-proof room. Freezing was scored and analyzed automatically using a CCD camera-based system (TimeFZ4, O’ Hara and Co., Ltd). Images were recorded from the top of each chamber using a camera at two frames per second. For the analysis of images, the gap area (pixel) between the contour of the mouse in one frame and that in the next frame was identified. Freezing behavior was considered to occur if the gap area was under 20 pixels for two continuous seconds. Freezing scores were expressed as the ratio of the freezing period to the experimental period. The discrimination index was calculated using the following formula: (freezing % in context A)/(average of freezing % in context B).

### Immunohistochemistry

Brains were fixed with 4% paraformaldehyde in phosphate-buffered saline (PBS) at 4°C overnight and sectioned at a thickness of 40 μm using a vibratome (Leica). Free-floating slices were permeabilized with 0.15% Triton X-100 in 5% bovine serum albumin (BSA)/PBS at room temperature for 30 min and then rinsed with PBS. Permeabilized slices were incubated with primary antibodies (rabbit anti-ZIF antibody, Santa Cruz Biotechnology; mouse anti-β-Galactosidase antibody, Promega) at 4°C overnight. Next, the slices were rinsed with PBS three times for 10 min and incubated with secondary antibodies (goat anti-rabbit Alexa Fluor 594, Molecular Probes; goat anti-mouse Alexa Fluor 488, Molecular Probes) at 4°C overnight. The slices were then rinsed with PBS for 10 min and subsequently incubated with 4′,6-diamidino-2-phenylindole (DAPI, Molecular Probes) at room temperature for 5 min. Next, the slices were rinsed with PBS three times for 10 min and mounted on a glass slide with Prolong Gold antifade reagent (Molecular Probes). For the quantification of immunoreactive cells, fluorescent images were acquired using a FV1000 confocal laser scanning microscope (Olympus), using a 40× objective lens at a speed of 12.5 μs per pixel by sequential illumination with UV (405 nm), Argon (488 nm) and HeNe (559 nm) lasers.

### Image Analysis

Three to four images were analyzed using the ImageJ software (NIH) for each animal in each region. Structures of ROI were anatomically defined according to The Mouse Brain in Stereotaxic Coordinates (Franklin and Paxinos, 2008; see Figure 3E). Along the anteroposterior axis, sections between level of −1.70 mm and −2.06 mm from the bregma were selected for the analysis of the dorsal hippocampus and the somatosensory cortex (SS). Along the mediolateral axis of the dorsal hippocampal CA1, three fixed positions comprising proximal and distal part were equally selected for imaging. As for the dorsal dentate gyrus (DG), four fixed positions comprising upper and lower blade were equally selected for imaging. As for the SS, three fixed positions comprising all layers were equally selected for imaging. Acquired 16-bit images were converted to 8-bit images, then threshold level was set to a range of 40–150 for the tau-lacZ immunoreactive images. As for ZIF immunoreactive images, threshold was set to a range of 70–255 (DG), 80–150 (CA1) and 70–255 (SS). The number of cells expressing DAPI, tau-lacZ or ZIF was quantified manually using the thresholded images. All quantification was performed blind to experimental conditions. We calculated double and chance levels as follows: double = (total number of cells co-labeled with tau-lacZ and ZIF)/(total number of DAPI positive cells); chance level = (total number of tau-lacZ positive cells/total number of DAPI positive cells) × (total number of ZIF positive cells/total number of DAPI positive cells) × 100. The double-to-chance ratio was calculated as double/chance level.

### Statistical Analysis

All data were statistically analyzed using Prism 5 (GraphPad software) and are presented as the mean ± SEM.

## Results

### Contextual Fear Memories Become Generalized Over Time

**Figure 1:**
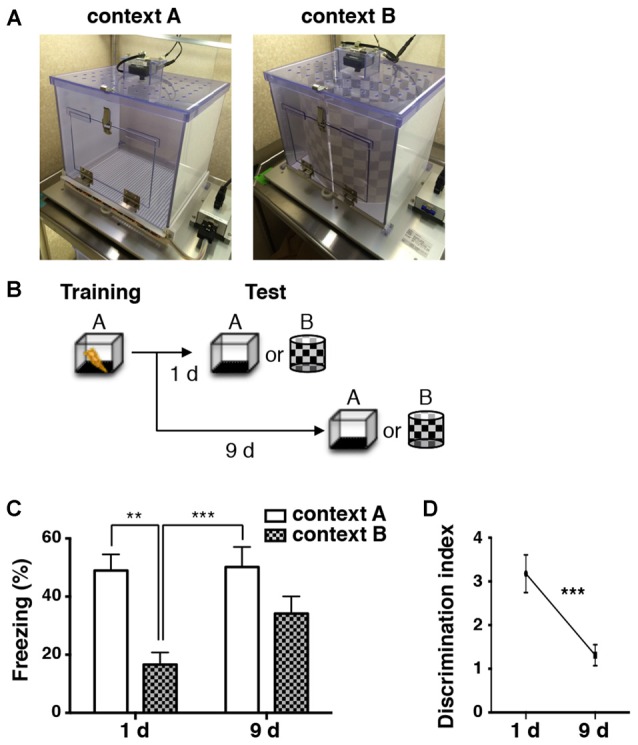
**Contextual fear memory is generalized over time. (A)** Images of chambers used in the fear conditioning experiments.** (B)** A schematic of the experimental design. Mice were trained for fear conditioning in context A. They were subjected to a memory retrieval test by exposure to either context A or context B 1 day (1d) and 9 days (9d) later. Different groups of animals were used for each test (1d: context A, *n* = 12; context B, *n* = 12. 9d: context A, *n* = 12; context B, *n* = 13). **(C)** Percentage of time spent frozen during the retrieval tests in conditioned context A (white bars) and in novel context B (checkered bars) at different time points. Two-way ANOVA followed by Tukey’s multiple comparisons test, **p < 0.01, ***p < 0.001. **(D)** Levels of the discrimination index at different time points after conditioning. Discrimination of context A and context B was significantly decreased over 9 days after fear conditioning. Unpaired *t*-test, ***p < 0.001.

During contextual fear conditioning, animals learn to associate a neutral context with an aversive event, such as foot shocks. Contextual fear memory is often generalized over the passage of time (Houston et al., 1999; Biedenkapp and Rudy, 2007; Wiltgen and Silva, 2007). To corroborate this phenomenon in our experimental condition, mice were subjected to electrical foot shocks in a training chamber (context A: Figures 1A,B) to elicit a long-term contextual fear memory. Then, they were placed in a conditioned chamber (context A) or a novel chamber (context B: Figures 1A,B) at 1 or 9 days after conditioning, and their freezing scores were measured as an index of contextual memory retrieval (Figure 1C). Naive cohorts of animals were used for each test to avoid any experience-related influences. The freezing scores of the retrieval test were significantly higher in context A compared to context B at 1 day after training (two-way ANOVA: main effect of context, *F*_(1,46)_ = 17.450, *p* < 0.001; Tukey’s *post hoc* test: *p* = 0.002; Figure 1C). However, at 9 days, exposure to both context A and context B evoked robust freezing, and there was no significant difference between the contexts (two-way ANOVA followed by Tukey’s *post hoc* test: *p* = 0.204; Figure 1C). In addition, the analysis of discrimination index showed significant differences among the time points (unpaired *t*-test: *p* < 0.001; Figure 1D). These results suggest that the contextual fear memories in our experimental condition were initially specific to the context, but became less specific within 9 days.

### Visualization of Neuronal Ensembles Activated During Encoding and Retrieval of Memory

**Figure 2:**
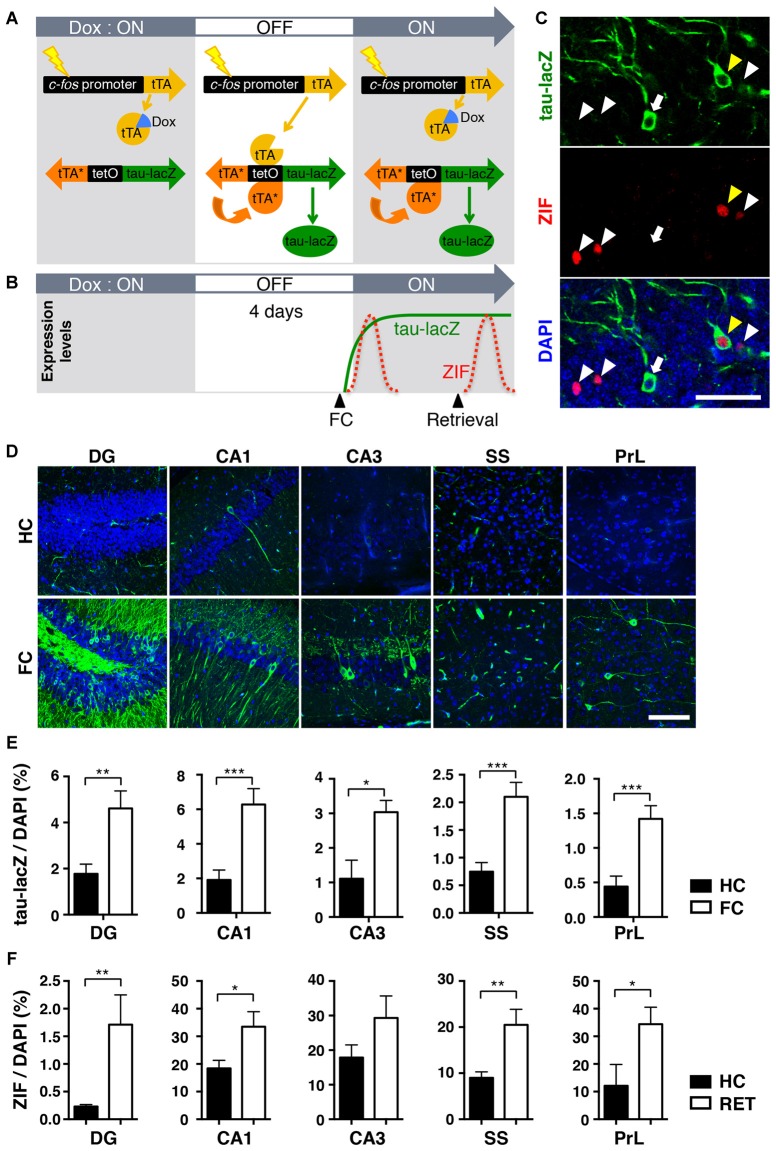
**The transgenic system used to label neuronal ensembles activated during the acquisition and retrieval of memory. (A)** Schematic representation of the transgenic system. When neuronal activity induces activation of the *c-fos* promoter in the absence of doxycycline (Dox), transactivator (tTA) drives the tetO promoter and induces the expression of tau-lacZ and the mutant tTA^H100Y^ (tTA*). Because tTA* can activate the tetO promoter even in the presence of Dox, neurons once activated during the off-Dox time window will continue to express tau-lacZ. **(B)** Schematic time-course representation of the genetic tau-lacZ (green line) and the endogenous ZIF (red dotted lines) expressions induced by two separated events. Neurons activated in response to fear conditioning during off-Dox expressed tau-lacZ persistently and endogenous ZIF transiently. Neurons activated by the retrieval test expressed endogenous ZIF, but the tetO-dependent tau-lacZ is not newly synthesized due to the presence of Dox. **(C)** Representative fluorescent confocal images showing cells labeled with tau-lacZ (green), ZIF (red) and 4′,6-diamidino-2-phenylindole (DAPI; blue) in the dentate gyrus (DG) of the transgenic mice. White arrows: neurons labeled only with tau-lacZ. White arrowheads: neurons labeled only with ZIF. Yellow arrowheads: neurons double-labeled with tau-lacZ and ZIF. Scale bar, 50 μm. **(D)** Representative fluorescent confocal images showing the expression of tau-lacZ (green) in various regions. Mice were either fear-conditioned (FC) or remained in the home cage (HC) after Dox removal. Scale bar, 100 μm. **(E)** The proportion of tau-lacZ positive cells in the dorsal DG (HC, *n* = 11; FC, *n* = 24), dorsal hippocampal CA1 (HC, *n* = 9; FC, *n* = 24) and CA3 (HC, *n* = 6; FC, *n* = 21), somatosensory cortex (SS; HC, *n* = 11; FC, *n* = 24), and prelimbic cortex (PrL; HC, *n* = 6; FC, *n* = 15). Black bars: HC, white bars: fear conditioned. Unpaired *t*-test: *p < 0.05, **p < 0.01, ***p < 0.001. **(F)** The proportion of ZIF positive cells in the dorsal DG (HC, *n* = 13; RET, *n* = 12), dorsal hippocampal CA1 (HC, *n* = 11; RET, *n* = 12) and CA3 (HC, *n* = 6; RET, *n* = 10), (SS; HC, *n* = 13; RET, *n* = 12), and (PrL; HC, *n* = 5; RET, *n* = 7). Black bars: HC, white bars: retrieved. Unpaired *t*-test: *p < 0.05, **p < 0.01.

The observed behavioral changes during memory retrieval tests over 9 days raised a possibility that different populations of cells might be activated during retrieval 1 day and 9 days after conditioning. To investigate this possibility, we visualized the specific neuronal ensembles activated in response to fear learning and retrieval in a single mouse brain. We used a tetracycline-regulated tTA transgenic system, in which neuronal activity induces activation of the *c-fos* promoter (Reijmers et al., 2007; Matsuo et al., 2008). In the absence of Dox, the tTA drives tetO promoter-linked tau-lacZ expression in those neurons selectively activated by behaviorally relevant events (Figure 2A). It also elicits the expression of the bi-directional tetO promoter-linked mutant tTA (tTA^H100Y^), which can trigger the tetO promoter even in the presence of Dox (Reijmers et al., 2007). Therefore, once the particular neurons are activated during a given time window in the absence of Dox, they can be labeled with tau-lacZ persistently due to the positive feedback loop system created by tTA^H100Y^ (Figures 2A,B; Reijmers et al., 2007).

To validate the induced expression of tau-lacZ in response to fear-conditioned (FC) learning, transgenic mice were trained with fear conditioning in context A after removing Dox for 4 days (Figure 2B). Soon after training, mice were fed with Dox diet to suppress further induction of tau-lacZ in neurons that were unrelated to the learning (Reijmers et al., 2007). A control group received the same Dox treatment, but the mice were kept in their HC. Immunohistochemical analysis revealed a sparse expression of tau-lacZ in the brain (Figures 2C,D), and the proportion of cells labeled with tau-lacZ was significantly larger in the FC mice 1 day after learning compared with the control HC group in the DG, the CA1 and CA3 regions of the dorsal hippocampus, the SS, and the prelimbic cortex (PrL; DG: *p* = 0.003; CA1: *p* < 0.001; CA3: *p* = 0.014; SS: *p* < 0.001; PrL: *p* < 0.001, unpaired *t*-test; Figure 2E).

When the trained animals are exposed to a conditioned chamber (context A) or a novel chamber (context B) after training, the neurons activated by memory retrieval should express endogenous immediate early genes (IEGs) such as *zif268* (ZIF; Saffen et al., 1988; Cole et al., 1989) 1–2 h later (Figures 2B,C,F). Thus, the previously activated neurons at the time of learning can be identified by long-lasting tau-lacZ expression, while neurons activated by retrieval would be labeled with ZIF within the same brain slice (Figures 2B,C). ZIF was used as an alternative neuronal activity marker due to the difficulty of discriminating endogenous c-Fos and transgenic c-Fos-EGFP (Reijmers et al., 2007; Matsuo et al., 2008) when we performed c-Fos immunohistochemistry. In this system, double-labeled cells should represent cells reactivated during the memory retrieval test.

### Reactivation of Neuronal Ensembles During Retrieval 1 Day After Learning

**Figure 3:**
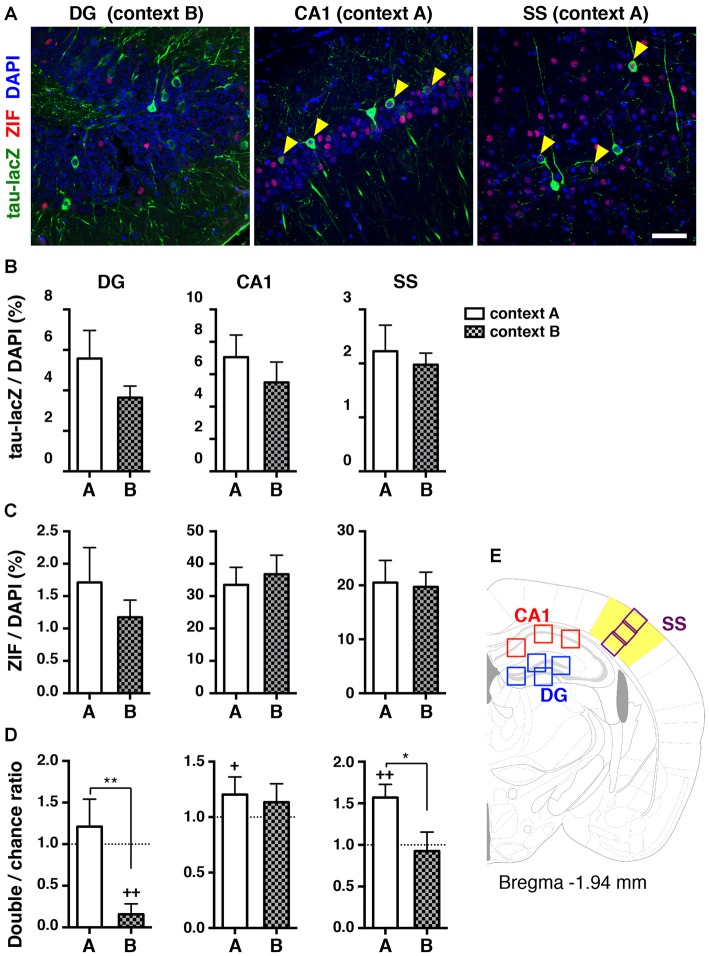
**Reactivation of cell ensembles during the retrieval test 1 day after learning. (A)** Representative confocal images of immunoreactive cells with tau-lacZ (green) and ZIF (red) in the dorsal DG, the dorsal hippocampal CA1, and the SS. The number of double-labeled cells in the DG was merely detected when mice were placed in context B 1 day after learning (left panel). A significant proportion of double-labeled cells (yellow arrowheads) was found in the CA1 and the SS when mice were re-exposed to context A 1 day after learning (middle and right panels, respectively). Blue: DAPI. Scale bar, 50 μm. **(B)** Quantification of tau-lacZ positive cells to DAPI positive cells in the DG, CA1 and SS.** (C)** Quantification of ZIF positive cells to DAPI positive cells in the DG, CA1 and SS. **(D)** Transgenic animals, which were tested for retrieval 1 day after learning in Figure 1, were used for the ensemble reactivation analysis. The percentage of neurons double-labeled with tau-lacZ and ZIF was compared to the chance level with the Wilcoxon signed rank test in the DG (context A, *n* = 12; context B, *n* = 12), the CA1 (context A, *n* = 12; context B, *n* = 12), and the SS (context A, *n* = 12; context B, *n* = 12). When the double:chance ratio equals 1, the probability that tau-lacZ positive cells overlap with ZIF positive cells is at the chance level. If the ratio is significantly larger than the chance level, neurons were significantly reactivated during retrieval. White bars: context A; checkered bars: context B. Wilcoxon signed rank test; ^+^*p* < 0.05, ^+^^+^*p* < 0.01. Mann Whitney test; *p < 0.05, **p < 0.01. **(E)** Schematic drawing of a mouse brain coronal section adapted from Franklin and Paxinos (2008), showing the regions of interest selected for measurements.

We analyzed the cells reactivated during the retrieval test 1 day after contextual fear conditioning, when animals did not show generalized fear memory. The proportion of tau-lacZ positive and ZIF positive cells was not significantly different between context A and context B in all regions analyzed (tau-lacZ; DG: *p* = 0.543, CA1: *p* = 0.343, SS: *p* = 0.917. ZIF; DG: *p* = 0.873, CA1: *p* = 0.602, SS: *p* = 0.960, unpaired *t-test*; Figures 3B,C). Then, the proportion of double-labeled cells was quantified to analyze the ratio of doubles to chance as an index of reactivation. In the dorsal hippocampal DG, the proportion of reactivated granule cells was at the chance level when mice were re-exposed to conditioned context A 1 day after learning (*p* = 1.000, Wilcoxon signed rank test; Figure 3D, Table 1). Remarkably, we found that the proportion of reactivated DG cells was significantly below the chance level in the mice that were placed in a novel context (context B) for the retrieval test 1 day after conditioning (*p* = 0.001, Wilcoxon signed rank test; Figures 3A,D). These findings suggest that different neuronal populations in the DG were positively recruited for contextual representations when mice were exposed to a distinct context that they had not previously encountered.

**Table 1:** **The number of double positive cells per 4′,6-diamidino-2-phenylindole (DAPI)-positive cells for each group**.

	DG	CA1	SS
1d A	14/25,427	214/9,577	51/8,965
1d B	2/23,562	173/7,470	25/8,100
9d A	11/35,082	76/7,831	37/9,570
9d B	7/30,254	43/8,072	19/8,777
9d C	4/52,147	120/16,402	10/8,888

In contrast to the DG cells, the proportion of reactivated dorsal CA1 pyramidal cells was not significantly different from the chance level when they were exposed to a distinct context B (*p* = 0.677, Wilcoxon signed rank test; Figure 3D). However, the proportion of reactivated CA1 pyramidal cells was significantly higher than the chance level when animals were returned to the same context 1 day after training (*p* = 0.041, Wilcoxon signed rank test; Figures 3A,D) though the reactivation indices were not significantly different between context A and context B (*p* = 0.443, Mann Whitney test). Several reports demonstrated that the same population of CA1 pyramidal cells activated during conditioning tended to be reactivated when the memory was retrieved in the trained context (Deng et al., 2013; Tayler et al., 2013).

Multimodal information, including sensory inputs, shapes contexts. We then examined the reactivation of neuronal ensembles in one of the primary sensory cortices, the SS. The proportion of reactivated cells in the cortex was significantly higher than the chance level in the mice re-exposed to context A at 1 day after learning (*p* = 0.001, Wilcoxon signed rank test; Figures 3A,D), whereas the percentage of reactivated cells during the retrieval test in context B was at the chance level (*p* = 0.910, Wilcoxon signed rank test; Figure 3D). Collectively, these results suggest that the hippocampal DG as well as the SS, were likely to distinguish the two different contexts at the ensemble activity level at the same time that the animals were able to discriminate the contextual differences at the behavioral level.

### Reactivation of Neuronal Ensembles During Retrieval 9 Days After Learning

**Figure 4:**
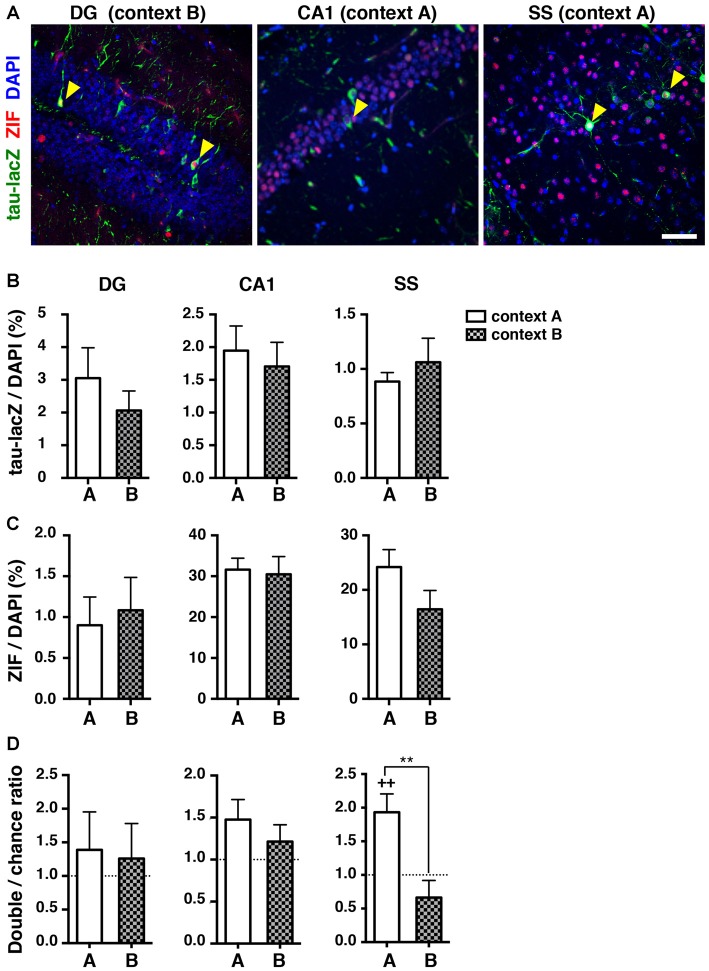
**Reactivation of cell ensembles during the retrieval test 9 days after learning. (A)** Representative confocal images of immunoreactive cells with tau-lacZ (green) and ZIF (red) in the dorsal DG, the dorsal hippocampal CA1, and the SS. Yellow arrowheads indicate double-labeled cells. Blue: DAPI. Scale bar, 50 μm.** (B)** Quantification of tau-lacZ positive cells to DAPI positive cells in the DG, CA1 and SS.** (C)** Quantification of ZIF positive cells to DAPI positive cells in the DG, CA1 and SS. **(D)** The percentage of neurons double-labeled with tau-lacZ and ZIF was compared to the chance level with the Wilcoxon signed rank test in the DG (context A, *n* = 12; context B, *n* = 13), the CA1 (context A, *n* = 12; context B, *n* = 12), and the SS (context A, *n* = 12; context B, *n* = 11). White bars: context A; checkered bars: context B. Wilcoxon signed rank test; ^+^^+^*p* < 0.01. Mann Whitney test; **p < 0.01.

Next, we examined the proportion of reactivated neuronal ensembles during the retrieval test 9 days after conditioning, when mice exhibited generalized fear memory. A substantial number of tau-lacZ labeled cells was detectable 9 days after training in the dorsal part of the hippocampal DG, the CA1 region, and the SS (Figures 4A,B), and it was comparable between context A and context B (DG: *p* = 0.758, CA1: *p* = 0.620, SS: *p* = 0.599, unpaired *t*-test; Figure 4B). Similarly, the proportion of ZIF positive cells was not significantly different between context A and context B (DG: *p* = 0.531, CA1: *p* = 0.495, SS: *p* = 0.103, unpaired *t*-test; Figure 4C). In the DG, the proportion of reactivated cells was not different from the chance level for both retrieval contexts (context A: *p* = 0.967; context B: *p* = 0.487, Wilcoxon signed rank test; Figures 4A,D, Table 1). Similarly, preferential reactivation of hippocampal CA1 neuronal ensembles was not observed in either the conditioned context A or the unconditioned context B (context A: *p* = 0.077; context B: *p* = 0.301, Wilcoxon signed rank test; Figures 4A,D). Moreover, the reactivation indices in the DG and CA1 were not significantly different between context A and context B (DG: *p* = 0.977; CA1: *p* = 0.298, Mann Whitney test; Figure 4D). These findings suggest that neuronal ensembles in the hippocampus were not specifically reactivated to the context during the retrieval test at this time point.

Unlike the hippocampus, the proportion of reactivated cells in the SS was significantly higher than the chance level in the mice re-exposed to context A at 9 days after learning (*p* = 0.009, Wilcoxon signed rank test; Figures 4A,D), whereas the percentage of reactivated cells during the retrieval test in context B was at the chance level (*p* = 0.198, Wilcoxon signed rank test; Figure 4D). These results indicate that a similar set of ensembles in the SS was preferentially reactivated when the mice were returned to the same environment, even though the animals appeared to be unable to distinguish the contexts at the level of behavioral output.

### Reactivation of Cell Ensembles During Memory Retrieval in a Context Where Animals do not Exhibit Generalized Fear at Later Time Point

**Figure 5:**
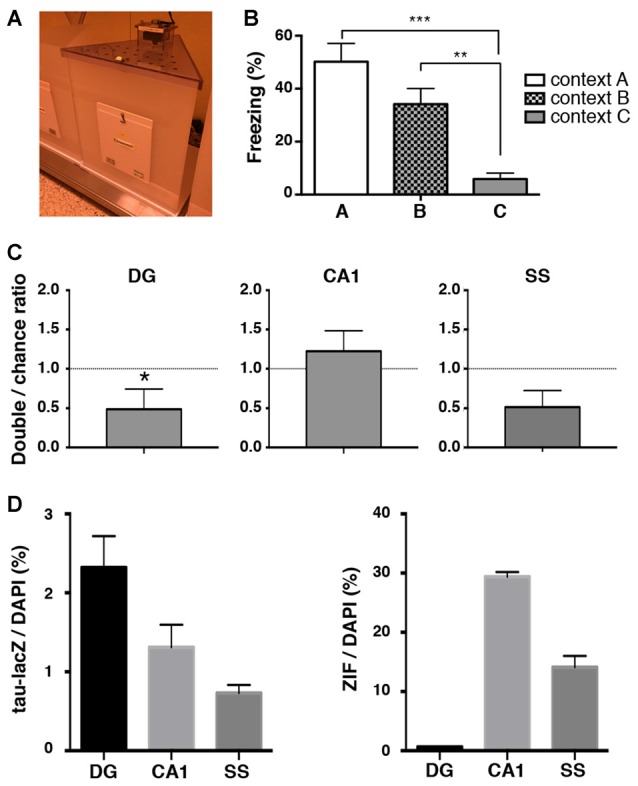
**Reactivation analysis in a novel context where mice do not exhibit generalized fear memory. (A)** Image of chamber used in the experiment. **(B)** Percentage of time spent frozen during the retrieval tests in conditioned context A (white bar, *n* = 12) and in context B (checkered bar, *n* = 13) and in context C (gray bar, *n* = 9) at 9 days after training. The same data from Figure 1C are used for the freezing percentage in context A and in context B. One-way ANOVA followed by Tukey’s multiple comparisons test. **p < 0.01, ***p < 0.001 **(C)** The percentage of neurons double-labeled with tau-lacZ and ZIF was compared to the chance level with the Wilcoxon signed rank test in the DG (*n* = 9), the CA1 (*n* = 9), and the SS (*n* = 9). The proportion of reactivated DG cells was significantly below the chance level. *p < 0.05. **(D)** Quantification of tau-lacZ and ZIF positive cells to DAPI positive cells in the DG, CA1 and SS.

Although we found a loss of unique reactivation pattern of DG neurons during retrieval 9 days after conditioning, the reduced number of tau-lacZ labeled cells at this time point raised a possibility that the result was an artifact of biased reduction of the labeling. Thus, we further analyzed the reactivation pattern of neuronal ensembles in a different novel context (context C: Figure 5A) where animals showed no significant freezing even 9 days after fear conditioning (one-way ANOVA: *F*_(2,32)_ = 12.67, *p* < 0.001; Tukey’s *post hoc* test, A vs. C: *p* < 0.001, B vs. C: *p* = 0.008; Figure 5B). In this condition, the proportion of reactivated DG cells was significantly below the chance level in the mice that were placed in context C (*p* = 0.031, Wilcoxon signed rank test), while the proportions in the hippocampal CA1 area and the SS were both at the chance level (CA1: *p* = 0.625, SS: *p* = 0.117, Wilcoxon signed rank test; Figures 5C,D). These results are not consistent with the reactivation pattern observed in context B at the same time point (9 days after conditioning), suggesting that the loss of DG ensemble reactivation pattern was not due to an artifact of reduced tau-lacZ labeling over days but rather an alteration of DG ensemble activities over time.

## Discussion

To understand the neural substrates mediating memory generalization, we investigated the specific neuronal ensembles activated during the acquisition and retrieval of a contextual fear memory. We used a transgenic mouse system that allowed us to examine the spatial patterns of active neurons at two different time points in a single mouse brain, with single-cell resolution. In this way, we identified unique reactivation patterns of neuronal ensembles in the hippocampal DG, and in the SS, during contextual fear memory retrieval. Notably, the unique reactivation pattern in the DG, but not in the SS, disappeared when the mice exhibited generalized fear memory.

The DG is postulated to play a crucial role in reducing the interference of redundant information, and in discriminating similar spatial or contextual inputs (pattern separation; O’Reilly and McClelland, 1994; Gilbert et al., 2001; McHugh et al., 2007). Our study showed that the population of reactivated DG cells was significantly less than the chance level when mice were placed in a novel context, which implicates a mechanism for the preferential recruitment of a distinct population of DG granule cells to represent a novel environment (Deng et al., 2013). The exclusively separated pattern of reactivation to a different context may represent a neural basis for pattern separation. Remarkably, this distinctive reactivation pattern disappeared when the mice exhibited generalization. A recent report demonstrated that DG granule cells selective α5-containing GABA_A_ receptor knock-out mice showed reduced tonic inhibition of the granule cells, and showed increased c-Fos positive cells when exposed to a novel environment (Engin et al., 2015). Interestingly, the mice had a deficiency in discriminating between two highly similar contexts (Engin et al., 2015). Such a molecular/cellular mechanism, which maintains sparseness of neuronal activation in the DG, could lead to a reduced interference between different contextual representations. Alternatively, neuronal competition-like mechanisms might play a crucial role in allocating different memories to distinct neuronal ensembles (Han et al., 2007; Matsuo, 2015; Stefanelli et al., 2016).

In contrast to the DG ensembles, we were not able to detect a significant difference of CA1 reactivation rate between conditioned context (context A) and novel context (context B) 1 day after conditioning although previous similar studies showed a significant difference (Deng et al., 2013; Tayler et al., 2013). There are several possibilities to explain this discrepancy. One possibility is that the differences between context A and context B in our study were not large enough so that the CA1 ensemble cannot discriminate. A previous report suggested that DG ensembles were able to detect small changes of contextual inputs while the CA1 ensembles were not (Deng et al., 2013). Alternatively, the high level of ZIF-positive neurons in the CA1 could mask a discriminatory activation pattern.

We cannot exclude the possibility that the reduced number of tau-lacZ labeled cells over time could affect the overlap measurements at 9 days after conditioning in the current study. For example, it is possible that the cells that are no longer lacZ labeled at 9 days may be those cells that express Zif post-retrieval, and that the overlap ratio calculated becomes lower than it should be. However, the overlap in the DG was rather increased when animals were put in the generalized context B, suggesting that the reactivation pattern was altered in DG cells over time. Moreover, if the overlap observed at 9 days was an artifact, the same tendency of reactivation should be expected irrespective of behavioral conditions. However, this was not the case. The reactivation ratio in the DG was significantly lower in the context C where animals did not show generalized fear memory while the ratio was chance level in the generalized context B at the same time point. A recent study using Arc promoter-mediated tagging system showed a differential reactivation of DG cells at recent and remote memory retrieval (Denny et al., 2014), supporting our results. But, usage of more reliable long-lasting tagging system is preferable for further studies. In our current study and previous reports (Reijmers et al., 2007; Deng et al., 2013; Cai et al., 2016), instead of endogenous c-Fos, ZIF was used as an activity marker of neurons activated during retrieval test. Thus, the proportion of reactivation rate could be different when endogenous c-Fos was used for the quantification.

In contrast to the irrelevant reactivation of DG ensembles during the retrieval test 9 days after learning, our data revealed a consistently preferential reactivation of neuronal ensembles in the SS when the mice were returned to the same context. This was true even after generalization had occurred at the behavioral level. These data suggest that the specific ensemble corresponding to a certain type of tactile stimulation has been predetermined during network formation in the SS, and the same ensemble is always activated in response to the same tactile stimuli. Alternatively, the cortical ensemble developed during initial learning had been maintained over 9 days. Offline reactivation of sequences of neuronal ensembles within the SS (Hoffman and McNaughton, 2002) may underlie the persistence of the original trace of tactile information. Early tagging of cortical networks upon encoding has been shown to be necessary for enduring memory formation (Lesburguères et al., 2011), and optogenetic reactivation of the cortical ensemble engaged by contextual fear conditioning has been shown to be sufficient for the retrieval of memory (Cowansage et al., 2014). However, the involvement of primary sensory cortex has not yet been demonstrated. Some studies demonstrated that inactivation of the hippocampus did not inhibit the precision of remote contextual memory (Wang et al., 2009; Kitamura et al., 2012). It is possible that the maintained ensemble activity in the primary sensory cortices or other cortical structures could spare precise discrimination in cases when hippocampal function is compromised.

Our results also suggest that the observed memory generalization at 9 days after learning does not reflect a dysfunction of sensory representation or processing in the neocortex, supporting an idea that generalization is not merely a failure in perceptual discrimination (Guttman and Kalish, 1956; Onat and Büchel, 2015). Tactile information reaches the hippocampus via the SS (Pereira et al., 2007; Bellistri et al., 2013). Thus, alteration in the ensemble representations within the hippocampus, or in upstream structures that link the sensory cortex to the hippocampus, likely account for memory generalization. The nucleus reuniens, which projects strongly into the hippocampus (Wouterlood et al., 1990), is one such candidate region to modulate the activity pattern of hippocampal neuronal ensembles. In fact, a recent optogenetic study reported that the activity of the nucleus reuniens during acquisition affected the specificity of contextual memory (Xu and Südhof, 2013). Interestingly, manipulation of the nucleus reuniens during memory retrieval had no effect on memory generalization. Because alterations of the hippocampal neuronal ensembles were detected during memory retrieval at 9 days, but not at 1 day, after memory acquisition in our experiments, a distinct circuit or mechanism may contribute to memory generalization that can be triggered during acquisition and retrieval.

The mechanism by which memories are generalized over time remains unclear. One plausible mechanism may involve a transition of contextual memory dependency, from the hippocampus to the neocortex, via system consolidation with passing time. Contextual fear memory retrieval shortly after conditioning depends on the hippocampus, and becomes independent of the hippocampus at a remote time point (Kim and Fanselow, 1992; Frankland and Bontempi, 2005; also see Goshen et al., 2011). Regarding the precision of remote memory, however, the role of the hippocampus is controversial. Some previous studies have reported that the hippocampus is always required for the retrieval of precise contextual memories (Winocur et al., 2007; Wiltgen et al., 2010), while a few other studies have indicated that the hippocampus is not required for precision remote memory retrieval (Wang et al., 2009; Kitamura et al., 2012). Our data support the former idea if the observed generalization stems from the loss of context-specific reactivation patterns of the hippocampal ensemble at 9 days after conditioning. However, associated fear influences the precision of contextual memory (Houston et al., 1999; Kitamura et al., 2012), so traumatic stress may affect the hippocampal ensemble activities and thereby contribute to a generalization of contextual fear memory (Besnard and Sahay, 2016). A combination of structural and functional imaging techniques, neuronal activity manipulation, and molecular analyses that focus on the specific neuronal ensemble representing a distinct fear memory, would enormously contribute to the understanding of pathophysiology of PTSD as well as the neural basis of fear learning.

## Author Contributions

MY conducted the experiments, and analyzed data. NM designed and supervised the experiments, analyzed data and wrote the manuscript.

## Funding

This work was supported by Grants-in-Aid for Scientific Research on Innovative Areas “Memory dynamism” (to NM), the Strategic Research Program for Brain Science (to NM) from the Ministry of Education, Culture, Sports, Science and Technology in Japan, the Takeda Science Foundation (to NM), and Japan Science and Technology Agency, PRESTO (to NM).

## Conflict of Interest Statement

The authors declare that the research was conducted in the absence of any commercial or financial relationships that could be construed as a potential conflict of interest. The reviewer HO declared a shared affiliation, though no other collaboration, with the authors to the handling Editor, who ensured that the process nevertheless met the standards of a fair and objective review.
